# Research on Joint-Angle Prediction Based on Artificial Neural Network for Above-Knee Amputees

**DOI:** 10.3390/s21217199

**Published:** 2021-10-29

**Authors:** Jianyu Yang, Guanchao Li, Xiaofei Zhao, Hualong Xie

**Affiliations:** Department of Mechanical Engineering and Automation, Northeastern University, Shenyang 110004, China; jyyang@mail.neu.edu.cn (J.Y.); lgc202109@163.com (G.L.); zhaoxiaofei5@hikvision.com (X.Z.)

**Keywords:** asymmetric lower extremity exoskeleton, electromyographic signals, artificial neural network, joint-angle prediction, going up and downstairs

## Abstract

In the current study, our research group proposed an asymmetric lower extremity exoskeleton to enable above-knee amputees to walk with a load. Due to the absence of shank and foot, the knee and ankle joint at the amputation side of the exoskeleton lack tracking targets, so it is difficult to realize the function of assisted walking when going up and downstairs. Currently, the use of lower-limb electromyography to predict the angles of lower limb joints has achieved remarkable results. However, the prediction effect was poor when only using electromyography from the thigh. Therefore, this paper introduces hip-angle and plantar pressure signals for improving prediction effect and puts forward a joint prediction method of knee- and ankle-joint angles by electromyography of the thigh, hip-joint angle, and plantar pressure signals. The generalized regression neural network optimized by the golden section method is used to predict the joint angles. Finally, the parameters (the maximum error, the Root-Mean-Square error (*RMSE*), and correlation coefficient (γ)) were calculated to verify the feasibility of the prediction method.

## 1. Introduction

The number of patients with lower-limb disabilities has been rapidly increasing. Most of them have lost their lower limbs due to natural disasters, traffic accidents and wars [1,2]. Currently, prostheses are the most significant tools to compensate for the walking function of lower-limb amputees [3,4,5,6,7]. However, most prostheses on the market, either passive or semiactive, cannot realize the joints’ active swinging, and thus fail to achieve assisted walking [8]. The lower extremity exoskeleton (LEE) assists amputees in rehabilitation training [9,10,11]. The LEE wearer can move around safely and flexibly for a long time at high speed under heavy load. The LEE has been widely used in marching, weight-bearing combat, and medical rehabilitation proposes [12,13].

**Figure 1:**
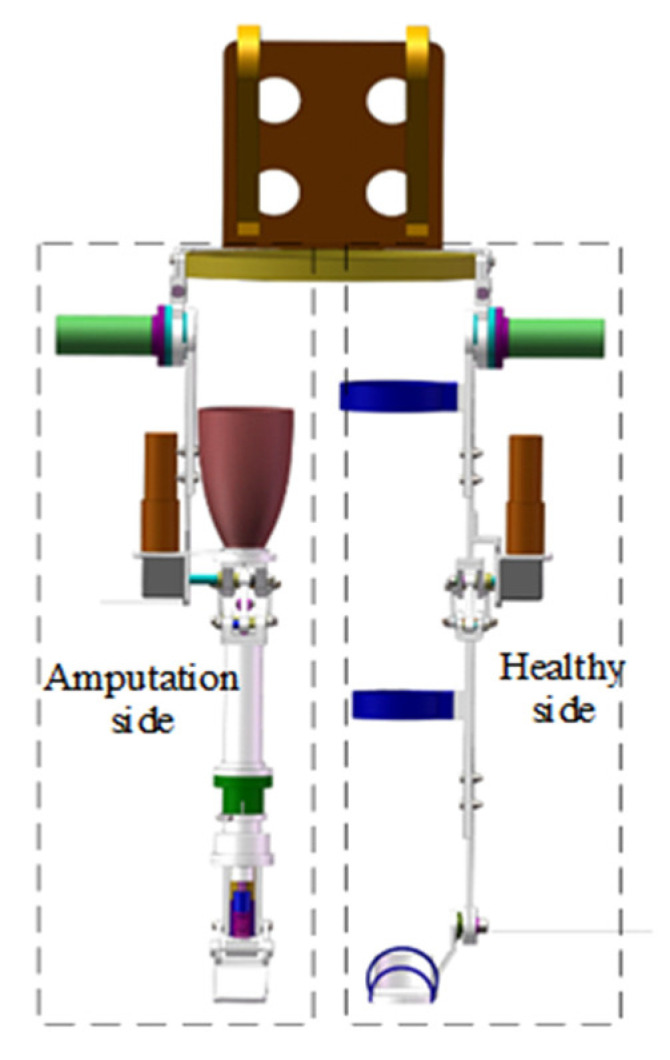
Asymmetric lower limb exoskeleton.

Considering the desire of above-knee amputees to walk like healthy persons and even walk with a load, as shown in Figure 1, our research group designs an asymmetric LEE [14] with two working modes, including the active mode and the semiactive mode. Under the active mode, the LEE motor provides a driving torque, enabling the wearer to walk with a load. When the prosthesis part is detached from the LEE, the prosthesis part can work as a normal prosthesis in the semiactive mode.

**Figure 2:**
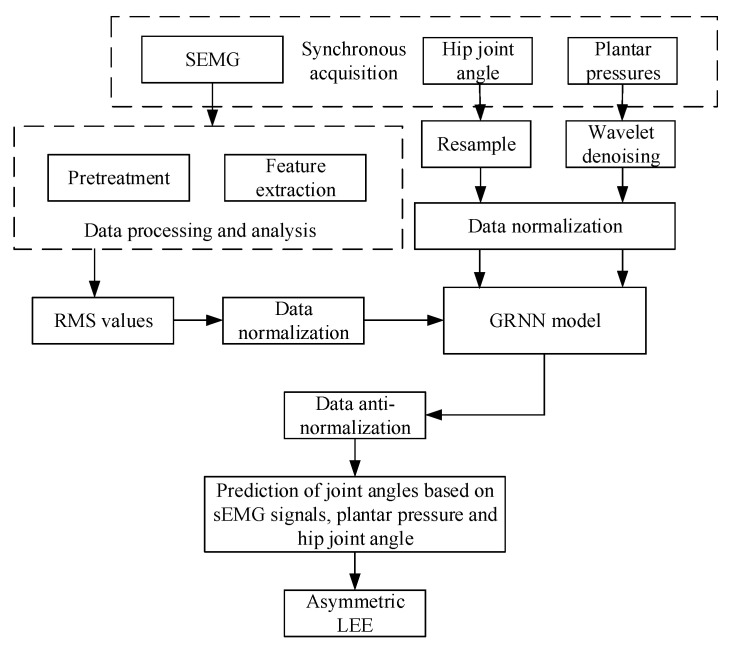
Flow chart of joint-angle prediction.

There are many problems that need to be solved in the research on asymmetric LEE. Additionally, this paper focuses on the study of how the prosthesis can better assist the patient to go upstairs and downstairs, when the prosthesis is disassembled from the LEE and worn on the patient’s residual limb to work alone. Therefore, it is important to obtain the joint angle of human lower limbs when controlling the prosthesis. Previous researchers have used electromyography (EMG) for joint-angle prediction. Chen et al. [15] and Zhang et al. [16] used the BP network to map the relationship between joint angles and EMG signals. Du et al. [17] used the least-squares extreme learning-machine algorithm based on the golden section to establish a nonlinear prediction model between surface electromyography (sEMG) and lower-limb joint angle. However, they used the EMG signals from the thigh and the calf. They did not evaluate the corresponding relationship between joint angles and EMG signals when walking up and downstairs, nor did they use the plantar pressure signals for the joint-angle prediction. Gaudet et al. [18] achieved the classification of upper limb phantom movements in transhumeral amputees using electromyographic and kinematic features. Previous scholars have proven that sEMG signals of residual limbs of amputation patients could be used for motion intention recognition, which verified the feasibility of sEMG signals in motion control for the prosthesis [19,20,21]. The muscles on the thigh stump could also be activated when amputee moves. The weak signals could be amplified and then be filtered, therefore the effective function could be obtained. Because of the lack of above-knee amputees to help with our research, the volunteers in this work were healthy people. A previous paper [22] demonstrates the feasibility and applicability of using healthy human body signals to study amputees. sEMG signals collected in the experiment were all from the muscles on the thigh related to the hip joint swing, to simulate the case of above-knee amputees as much as possible. In this study, we found that the existing prediction methods based on angle and sEMG signals are slightly less accurate during the support period. Considering that the plantar pressure signal is closely related to the support period and has the characteristics of periodic changes, this natural repeatability was exploited to improve the accuracy of the estimation method, which is the novelty of this paper. The research content is shown in Figure 2. This paper is organized as follows. Section 2 starts by the establishment of a data acquisition system, the collection and processing of sEMG, joint angle and plantar pressure signals. It ends with the design of an artificial neural network that focus on the angle estimation. The obtained results are presented and analyzed in Section 3, while Section 4 discusses them. Conclusions are presented in Section 5.

## 2. Experiment and Methods

### 2.1. Data Acquisition System Establishment

**Figure 3:**
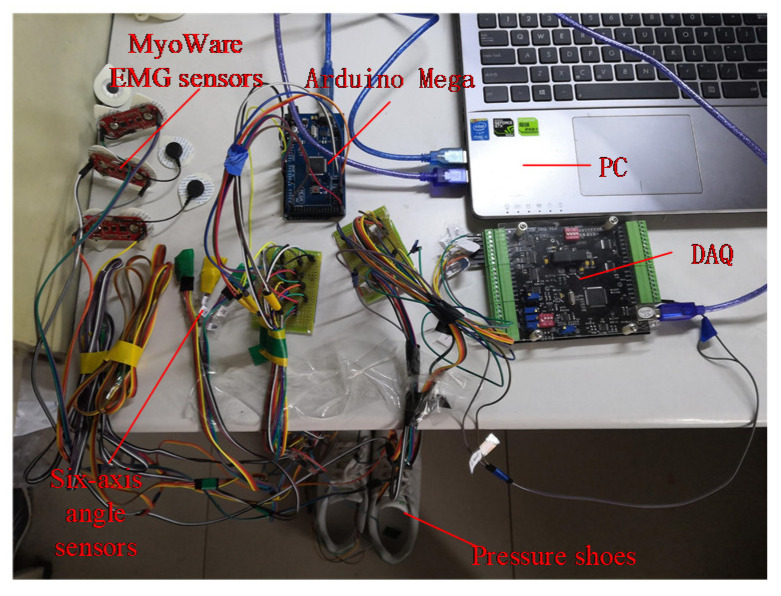
Designed data acquisition system.

**Figure 4:**
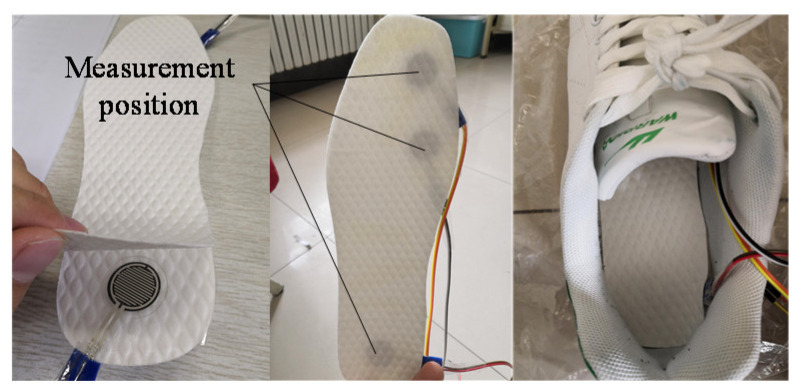
Position of pressure sensors.

To obtain EMG, joint angle, and plantar pressure signals synchronously, a data acquisition system was designed in this paper, as shown in Figure 3. This system was composed of thin-film pressure sensors, EMG sensors, six-axis angle sensors, a data acquisition card, an ARDUINO MEGA microcontroller, etc. To acquire the pressure signals under the big toe, forefoot, and heel in real-time, three thin-film pressure sensors were placed in an insole, as shown in Figure 4. Due to the limitation of sensors, different signals were collected by different devices. In order to ensure data synchronization, we figured out a way to solve the problem of synchronization. Angle signals and plantar pressure signals were collected by Arduino synchronously, while sEMG signals and plantar pressure signals were collected by the data acquisition card synchronously. During the data acquisition process, plantar pressure signals were the same. Pressure signal data were generated simultaneously although they were collected by different equipment. Based on the pressure signals, the angle and the sEMG signals could be time-synchronized. The frequency of sEMG collected by the data acquisition card was very stable. Additionally, the effective part of the sEMG signal was mainly distributed in the range of 0–500 Hz. According to the sampling theorem, the frequency is finally set as 2000 Hz. Band-pass filtering was performed on sEMG signals to remove interference, and the root-mean-square eigenvalues were extracted at every 20 data points. So, the final frequency of processed sEMG signals is 100 Hz. The frequency of raw plantar pressure is also 2000 Hz. Then, the averages of plantar pressure signals were extracted as the eigenvalues of pressure data every 20 points. After processing, the frequency of the pressure signal is 100 Hz. In the process of collecting angle signals, due to equipment problems, the sampling frequency of the angle signal was affected. In order to ensure that the frequency of the angle signal was consistent with the sEMG and pressure signal, the angle signal was first fitted and then resampled, and the final frequency was 100 Hz. As a result, angle, pressure, and sEMG signals could be frequency-synchronized.

### 2.2. Data Acquisition

**Figure 5:**
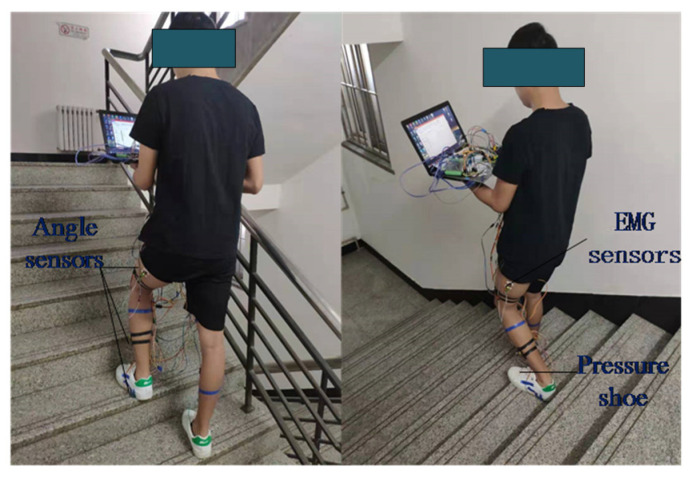
Data acquisition process.

The data in this paper were collected from three healthy males (aged 25 ± 2 years). Each one walked upstairs and downstairs 20 times. Before the experiment, no vigorous exercise was done by the technicians, and alcohol was used to clear the skin and increase the conductance. The EMG signals were measured in the rectus femoris, biceps femoris and semitendinosus muscles of the thigh. Furthermore, the angle signals and plantar pressure signals were simultaneously acquired. As examples, two data acquisition photos are shown in Figure 5.

### 2.3. Surface EMG Signal Preprocessing

**Figure 6:**
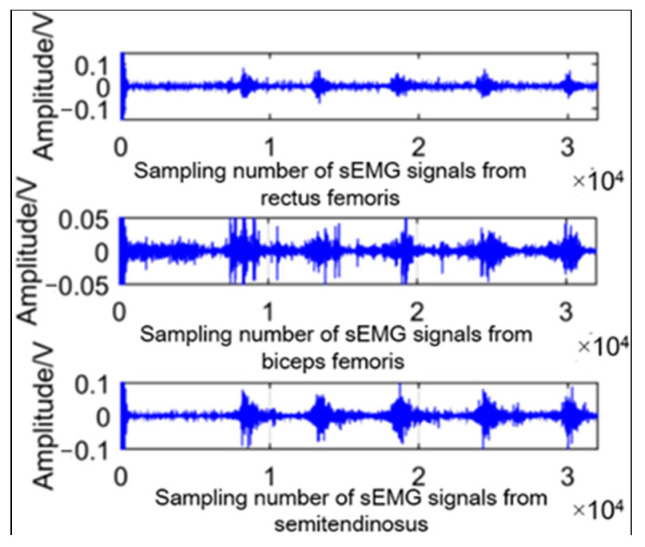
EMG signals in going upstairs procedure after band-pass filter.

According to the Nyquist sampling theorem, the sampling frequency of EMG signals in this paper was set at 2000 Hz. The raw EMG signals were then collected, and signals were filtered by a bandpass filter with a frequency range of 20–500 Hz. The parameters of filter is shown in Table 1. One of the filtered signals is shown in Figure 6.

**Figure 7:**
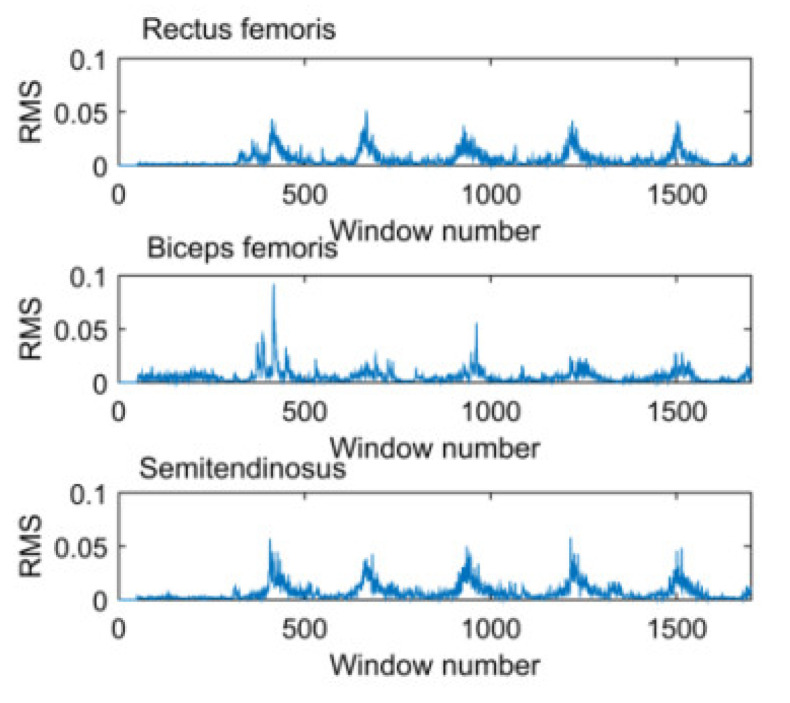
*RMS* of sEMG signals.

Next, the characteristic values of sEMG signals that were filtered were extracted. There are many characteristic values, we selected the most commonly used Root Mean Square (*RMS*) that could contain the most useful information in the signal, as shown in Figure 7. *RMS* values were calculated every 20 data points by:

(1) $$RMS=\sqrt{\frac{1}{N}\sum_{i=1}^{N}x{\left(i\right)}^{2}}$$

### 2.4. Joint-Angle Signal Processing

**Figure 8:**
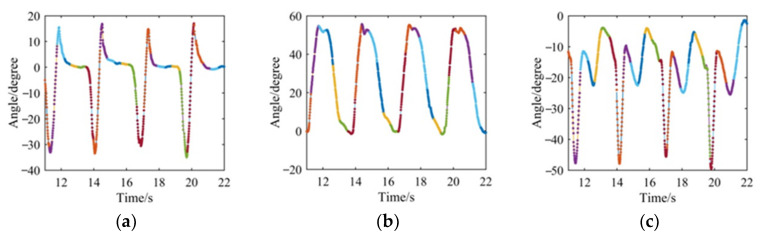
Piecewise curve-fitting results in going upstairs process. (**a**) Angle of hip joint; (**b**) Angle of knee joint; (**c**) Angle of ankle joint.

The angle signals were sampled twice to unite the sampling frequency of sEMG signals, so the final frequency of the angle was 100 Hz. In this paper, piecewise polynomial curve fitting was used for resampling of joint-angle signals. Figure 8 shows one of the piecewise curve-fitting results during going up the stairs.

### 2.5. Plantar Pressure Signal Processing

**Figure 9:**
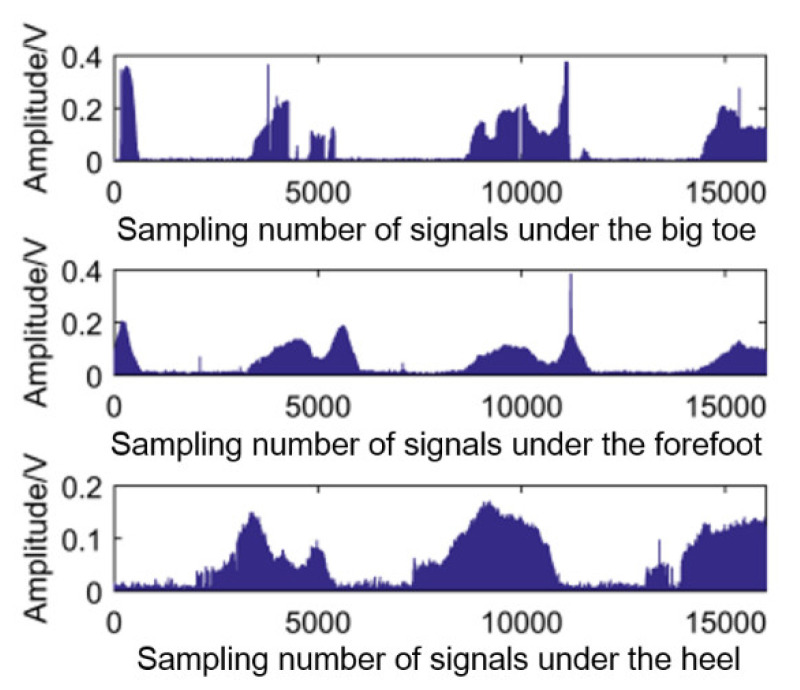
The raw plantar pressure signals.

**Figure 10:**
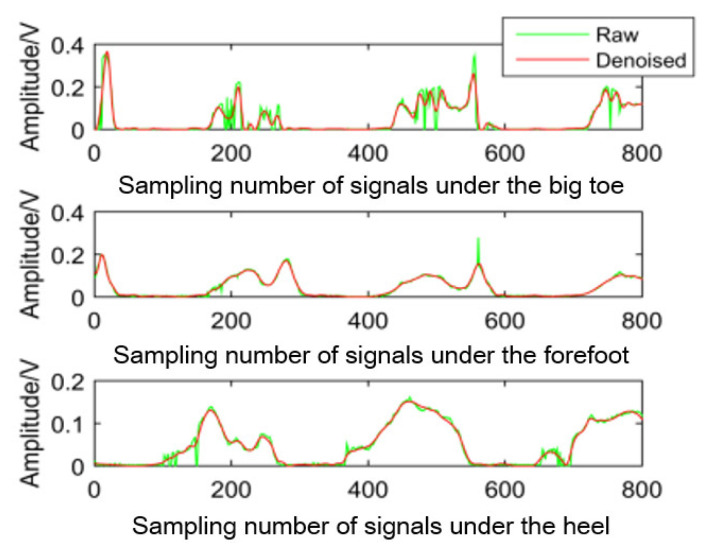
Characteristic values of plantar pressure signals.

The plantar pressure signals were processed by taking the time windows because the pressure signal and sEMG signals were collected synchronously. Each window has 20 points, and the average value of the data in each window is calculated as the characteristic value of pressure signals. The raw plantar pressure signals and the characteristic values are shown in Figure 9 and Figure 10, respectively.

### 2.6. GS-GRNN Network for Angle Estimation

**Figure 11:**
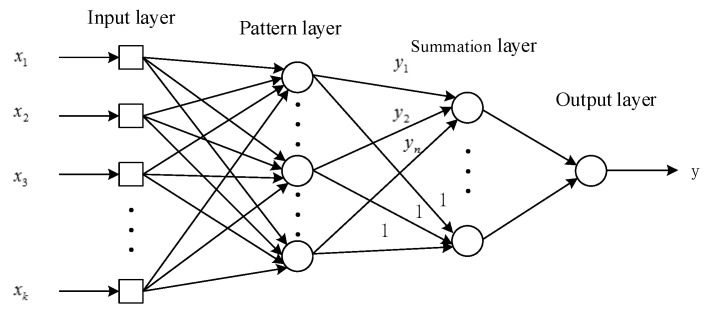
Structure of GRNN.

In the machine-learning algorithm, the support vector machine (SVM) performs efficiently in pattern recognition [23,24]; however, it is not effective in dealing with nonlinear mapping problems. This paper used an artificial neural network to map the joint angles [25,26,27]. Generalized regression neural network (GRNN) is a network developed from radial basis function neural network, as shown in Figure 11.

Suppose the joint probability density of random variable *x* and random variable *y* is *f* (*x*, *y*), and the observed value of *x* is *X*, then the regression of *y* relative to *X* is the prediction output of the neural network as follows:

(2) $$\hat{Y}(X)=\frac{\sum_{i=1}^{n}Y_{i}\exp[-\frac{{\left(X-X_{i}\right)}^{T}(X-X_{i})}{2\sigma^{2}}]}{\sum_{i=1}^{n}\exp[-\frac{{\left(X-X_{i}\right)}^{T}(X-X_{i})}{2\sigma^{2}}]}$$

where $\hat{Y}$ is the prediction result of *Y* under the condition that the input is *X*, *X_i_* and *Y_i_* are the observed values of *X* and *Y*, *n* is the number of samples, *σ* is the smoothing factor (*σ* > 0), $D_{i}^{2}={\left(X-X_{i}\right)}^{T}(X-X_{i})$ is the square of Euclidean distance between *X* and *X_i_*. The transfer function of the hidden layer is:

(3) $$p_{i}=\exp[-\frac{{\left(X-X_{i}\right)}^{T}(X-X_{i})}{2\sigma^{2}}]$$

The units in the summation layer are the denominator and numerator of Equation (2), respectively:

(4) $$S_{D}=\sum_{i=1}^{n}P_{i}$$

(5) $$S_{N}=\sum_{i=1}^{n}Y_{i}P_{i}$$

Therefore, the result of the output layer is:

(6) $$\hat{Y}=\frac{S_{N}}{S_{D}}$$

**Figure 12:**
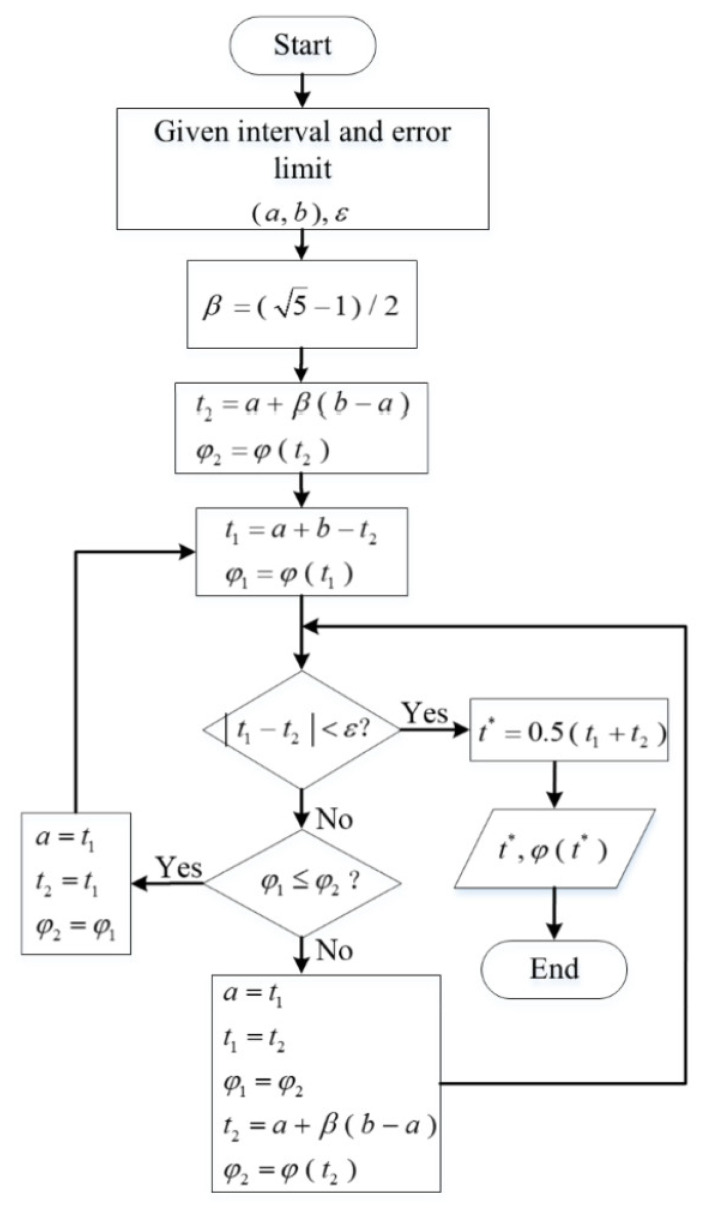
The flow chart of the golden section algorithm.

The smoothing factor affects the network performance, which has a significant influence on the network. In this paper, the generalized regression neural network was optimized by golden section algorithm (GS-GRNN). The objective function is the mean square error between the predicted value *Y_i_* of the joint angle and the measured value of *Y*:

(7) $$E=\frac{1}{N}\sum_{i=1}^{N}{\left(Y_{i}-Y\right)}^{2}$$

where *N* represents the number of predicted samples. The flow chart of the golden section algorithm is shown in Figure 12, where φ(t) is the objective function, ε is the termination limit, and σ is the value range of (*a*, *b*).

### 2.7. Data Normalization Processing

Before the training of an artificial neural network, the data was normalized to avoid singular sample data. In this paper, the data were normalized by:

(8) $$x^{\prime }=\frac{x-minA}{maxA-minA}$$

where x is the original data, $x^{\prime }$ is the corresponding value after normalization, maxA is the maximum value set, and minA is the minimum data set.

## 3. Analysis of Results

**Figure 13:**
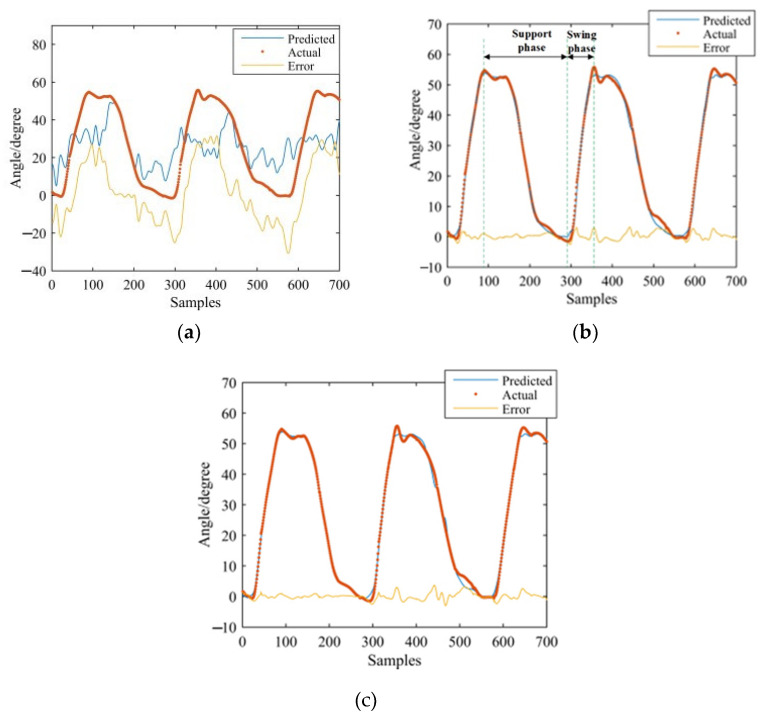
Prediction results of hip-joint angle in the going upstairs process. (**a**) Results of IN1; (**b**) Results of IN2; (**c**) Results of IN3.

**Figure 14:**
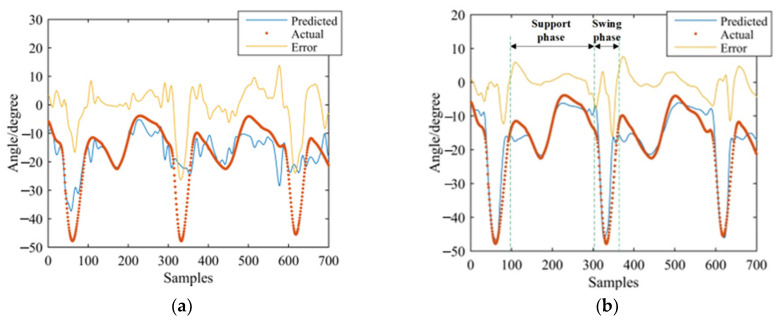

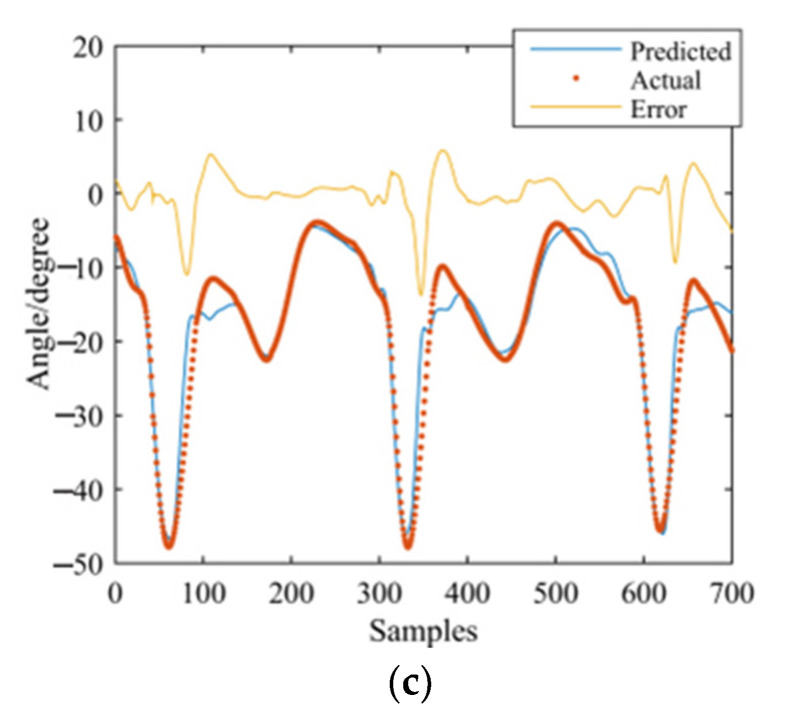
Prediction results of knee-joint angle in the going upstairs process. (**a**) Results of IN1; (**b**) Results of IN2; (**c**) Results of IN3.

**Figure 15:**
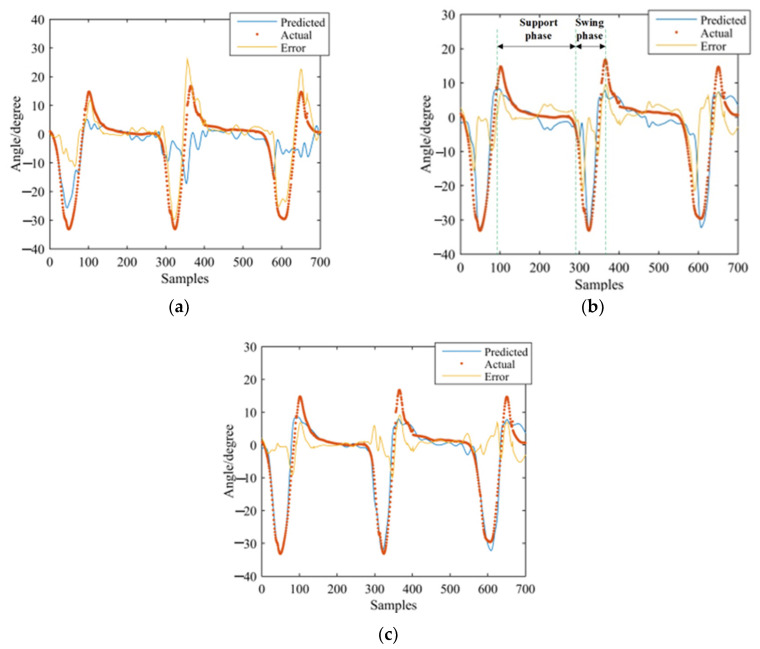
Prediction results of ankle-joint angle in the going upstairs process. (**a**) Results of IN1; (**b**) Results of IN2; (**c**) Results of IN3.

**Figure 16:**
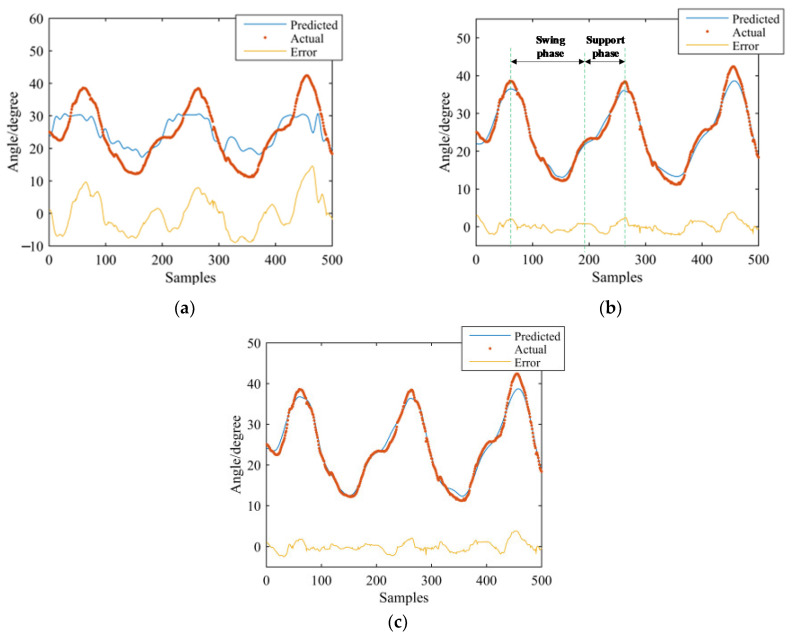
Prediction results of hip-joint angle in the going downstairs process. (**a**) Results of IN1; (**b**) Results of IN2; (**c**) Results of IN3.

**Figure 17:**
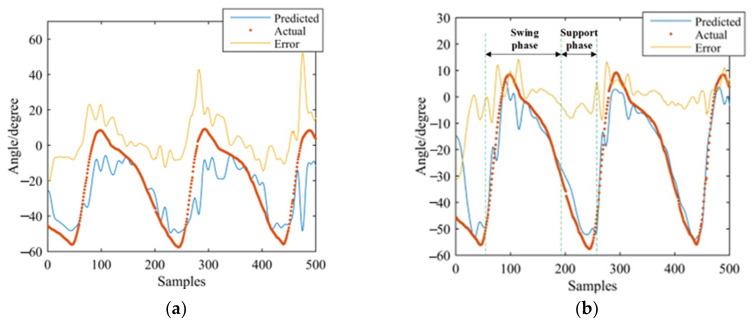

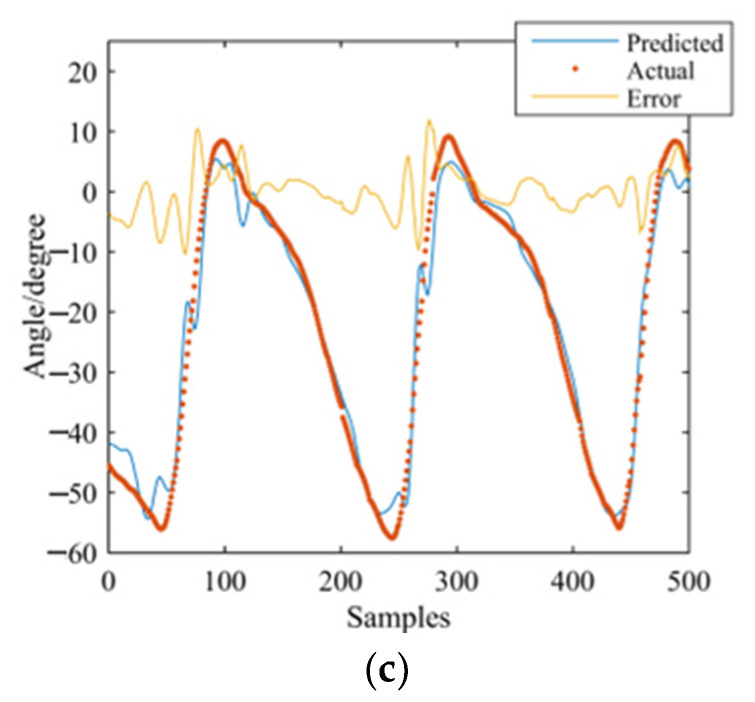
Prediction results of knee-joint angle in the going downstairs process. (**a**) Results of IN1; (**b**) Results of IN2; (**c**) Results of IN3.

**Figure 18:**
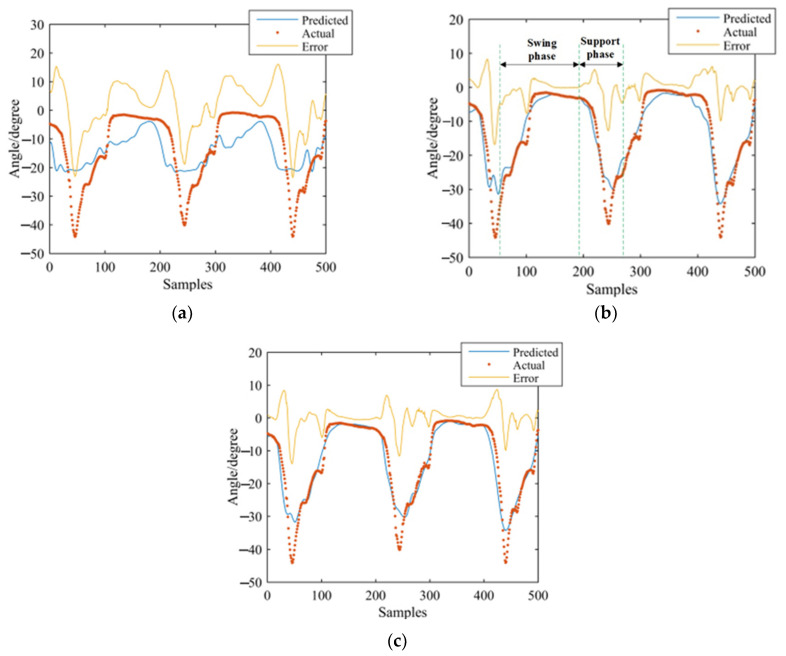
Prediction results of ankle-joint angle in the going downstairs process. (**a**) Results of IN1; (**b**) Results of IN2; (**c**) Results of IN3.

GS-GRNN was used to predict the joint angles during the going upstairs and downstairs processes. The inputs were EMG signals of the thigh (IN1), EMG signals and angle signals of the hip joint (IN2), EMG signals, angle signals of the hip joint and plantar pressure signals (IN3). The outputs were angle signals of the hip joint, knee joint, and ankle joint. Among them, one third of that data was used for validation and testing, and two thirds of that data was used for training. We take one set of experimental data during going upstairs and downstairs as examples. Figure 13, Figure 14, Figure 15, Figure 16, Figure 17 and Figure 18 show the prediction results of each joint angle.

**Figure 19:**
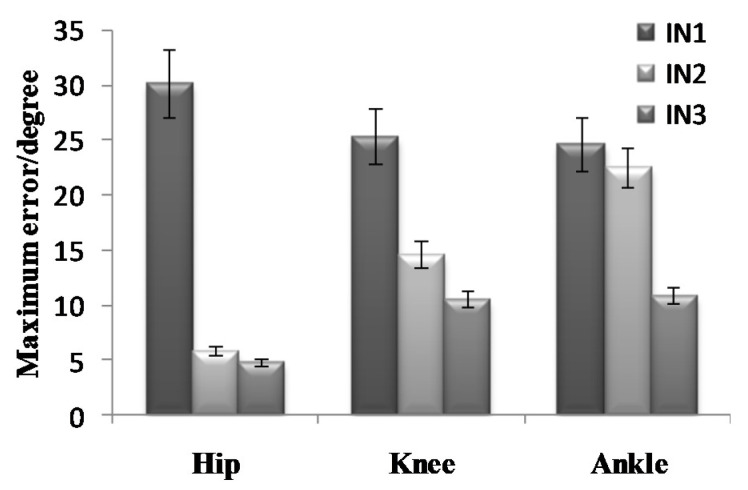
Maximum error of the prediction results in the going upstairs process.

**Figure 20:**
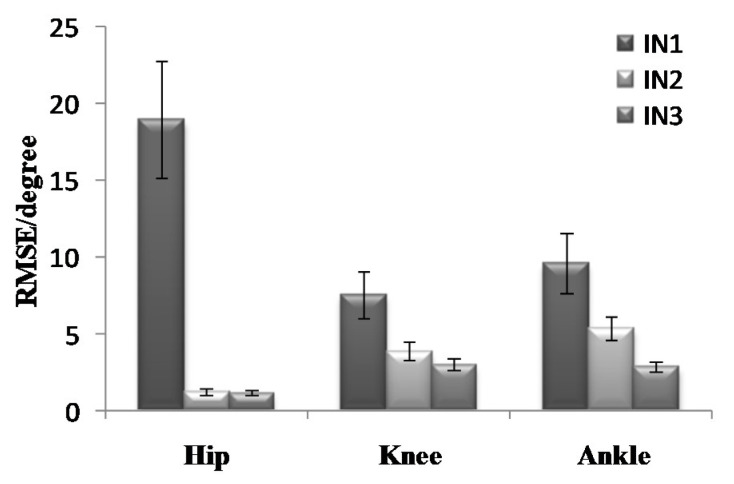
*RMSE* of the prediction results in the going upstairs process.

**Figure 21:**
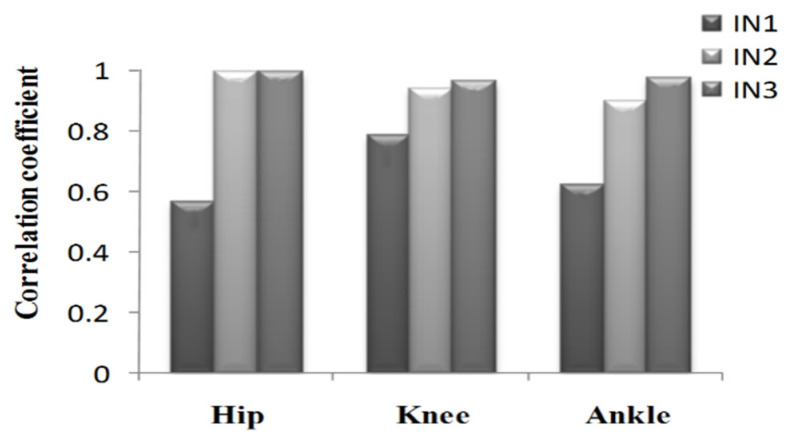
Correlation coefficient of the prediction results in the going upstairs process.

**Figure 22:**
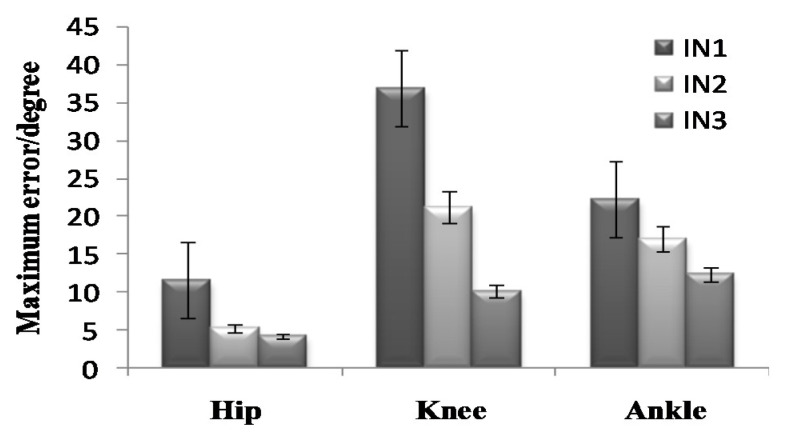
Maximum error of the prediction results in the going downstairs process.

**Figure 23:**
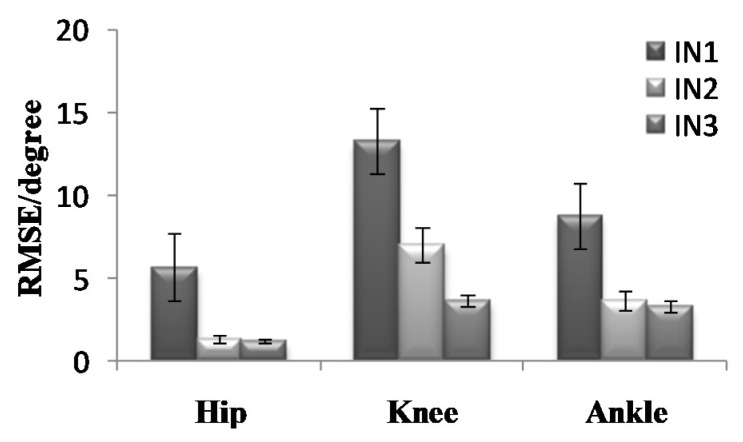
*RMSE* of the prediction results in the going downstairs process.

**Figure 24:**
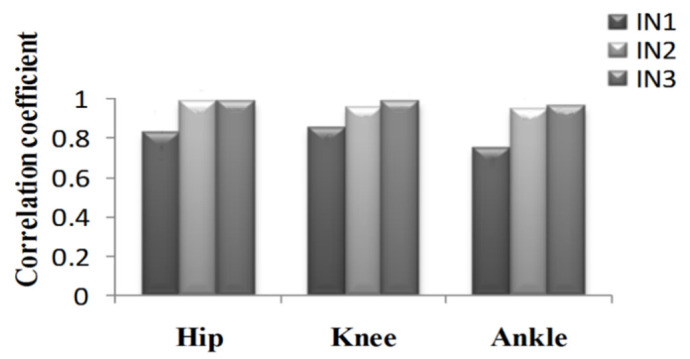
Correlation coefficient of the prediction results in the going downstairs process.

To further analyze the prediction results, the maximum error, the Root-Mean-Square error (*RMSE*), and correlation coefficient (γ) were calculated as shown in Figure 19, Figure 20, Figure 21, Figure 22, Figure 23 and Figure 24.

(9) $$RMSE=\sqrt{\frac{1}{N}\sum_{i=1}^{N}{\left(x_{i}-y_{i}\right)}^{2}}$$

(10) $$\gamma =\frac{\frac{1}{N}\sum_{i=1}^{N}\left(x_{i}-\bar{x}\right)(y_{i}-\bar{y})}{\sqrt{\frac{1}{N}\sum_{i=1}^{N}{\left(x_{i}-\bar{x}\right)}^{2}}\sqrt{\frac{1}{N_{a}}\sum_{i=1}^{N_{a}}{\left(y_{i}-\bar{y}\right)}^{2}}}$$

where, $x_{i}$ is the predicted value, $y_{i}$ is the actual value, N is the number of samples, $\bar{x}$ and $\bar{y}$ are the mean values of the predicted value, and the actual measured value, respectively.

## 4. Discussion

We found that the addition of plantar pressure signals can effectively improve the prediction effect of knee and ankle angles. The prediction effect was the best when imputing EMG signals, hip-joint angle signals, and plantar pressure signals synchronized with each other. During going upstairs, the correlation coefficients of the hip-, knee-, and ankle-joint angles can reach 0.9986, 0.9649 and 0.9771, respectively. The root-mean-square errors are 1.1530, 3.0077 and 2.8407, respectively. During going downstairs, the correlation coefficients can reach 0.9921, 0.9893 and 0.9635, and the root-means-square errors are 1.1725, 3.5974 and 3.3239, respectively. As discussed in the results above, based on the GS-GRNN network, lower-limb-joint angle prediction is realized in this paper. When only the hip-joint angle was used as an input, low accuracy and high error were achieved. When the input included angle and EMG signals, the accuracy and the correlation coefficients were significantly improved. When the angle, EMG, and plantar pressure signals were simultaneously imported into GRNN together, the accuracy and the correlation coefficient were further improved. The addition of plantar pressure signals expands the sample data. Generally, when the input signals and data samples increase, the neural network becomes more trained, and more accurate results could be obtained.

## 5. Conclusions

This paper optimizes the joint-angle prediction method by integrating hip-joint angle signals, sEMG signals, and plantar pressure signals to solve the problem of online gait planning of asymmetric lower extremity exoskeletons. Furthermore, each joint follows a periodic trajectory in the function of the gait phase. Therefore, the plantar pressure signal that contains gait phase information improves this work and enriches the motion information of the input data, improving the prediction accuracy.

GS-GRNN used for joint-angle prediction performs well in this paper. The prediction results confirmed the optimization method’s feasibility, and finally, a joint-angle prediction method for above-knee amputees was developed, which provided a favorable reference for LEE’s movement control.

To ensure the patients’ safety, we have been recently practicing these techniques on healthy people. However, with the development of research, we will try to recruit amputees so that we can to make this algorithm more adaptable.

## Figures and Tables

**Table 1:** Main parameters of band-pass filter.

Parameters	Value
Pass-band Upper Cutoff Frequency (Hz)	20
Pass-band Lower Cutoff Frequency (Hz)	500
Pass-band Maximum Attenuation (dB)	3
Stop-band Upper Cutoff Frequency (Hz)	10
Stop-band Lower Cutoff Frequency (Hz)	570
Stop-band Minimum Attenuation (dB)	20

## Data Availability

Not applicable.
